# Monkeypox in a Traveler Returning from Nigeria — Dallas, Texas, July 2021

**DOI:** 10.15585/mmwr.mm7114a1

**Published:** 2022-04-08

**Authors:** Agam K. Rao, Joann Schulte, Tai-Ho Chen, Christine M. Hughes, Whitni Davidson, Justin M. Neff, Mary Markarian, Kristin C. Delea, Suzanne Wada, Allison Liddell, Shane Alexander, Brittany Sunshine, Philip Huang, Heidi Threadgill Honza, Araceli Rey, Benjamin Monroe, Jeffrey Doty, Bryan Christensen, Lisa Delaney, Joel Massey, Michelle Waltenburg, Caroline A. Schrodt, David Kuhar, Panayampalli S. Satheshkumar, Ashley Kondas, Yu Li, Kimberly Wilkins, Kylie M. Sage, Yon Yu, Patricia Yu, Amanda Feldpausch, Jennifer McQuiston, Inger K. Damon, Andrea M. McCollum, July 2021 Monkeypox Response Team, July 2021 Monkeypox Response Team, Asma’u Aminu-Alhaji, Lauren Andersen, Matthew Arduino, Nicolette Bestul, Megan Bias, Mary J. Choi, Crystal Gigante, Madison Harkey, Kate Hendricks, Yonette Hercules, Farah Husain, Oladipupo Ipadeola, Robynne Jungerman, Theodora Khan, Grishma Kharod, Amber Kunkel, Amanda MacGurn, Audrey Matheny, Timothy McCleod, Faisal S. Minhaj, Jenna Mink, Clint Morgan, Yoshinori Nakazawa, Donovan Newton, Eddy Ortega, Lalita Priyamvada, Kay Radford, Joseph Rehfus, Muhammad Muhammad Saleh, Michael B. Townsend, Rita Traxler, Florence Whitehill, Xianfu Wu, Hui Zhao, Michelle Carruthers, Ivory Gomez, Samantha Groppell, Juan Jaramillo, Daniel Serinaldi, Jose Serrano, Joey Stringer, Jenna Gettings, Jessica Pavlick, José David Retana, Shelley Stonecipher, Rachael Straver, Inger-Marie Vilcins, Leisha D. Nolen

**Affiliations:** ^1^Division of High-Consequence Pathogens and Pathology, National Center for Emerging and Zoonotic Infectious Diseases, CDC; ^2^Dallas County Health and Human Services, Dallas, Texas; ^3^Division of Global Migration and Quarantine, National Center for Emerging and Zoonotic Infectious Diseases, CDC; ^4^Texas Health Presbyterian Hospital, Dallas, Texas; ^5^Division of Preparedness and Emerging Infections, National Center for Emerging and Zoonotic Infectious Diseases, CDC; ^6^Texas Department of State Health Services; ^7^Division of Healthcare Quality Promotion, National Center for Emerging and Zoonotic Infectious Diseases, CDC; ^8^National Institute for Occupational Safety and Health, CDC; ^9^Utah Department of Health; ^10^Georgia Department of Public Health.; CDC; CDC; CDC; CDC; CDC; CDC; CDC; CDC; CDC; CDC; CDC; CDC; CDC; CDC; CDC; CDC; CDC; CDC; CDC; CDC; CDC; CDC; CDC; CDC; CDC; CDC; CDC; CDC; CDC; CDC; CDC; CDC; CDC; CDC; Dallas County Health Department, Texas; Dallas County Health Department, Texas; Dallas County Health Department, Texas; Dallas County Health Department, Texas; Dallas County Health Department, Texas; Dallas County Health Department, Texas; Dallas County Health Department, Texas; Georgia Department of Public Health; Georgia Department of Public Health; Texas Department of State Health Services; Texas Department of State Health Services; Texas Department of State Health Services; Texas Department of State Health Services; Utah Department of Health.

Monkeypox is a rare, sometimes life-threatening zoonotic infection that occurs in west and central Africa. It is caused by *Monkeypox virus*, an orthopoxvirus similar to *Variola virus* (the causative agent of smallpox*)* and *Vaccinia virus* (the live virus component of orthopoxvirus vaccines) and can spread to humans. After 39 years without detection of human disease in Nigeria, an outbreak involving 118 confirmed cases was identified during 2017–2018 (*1*); sporadic cases continue to occur. During September 2018–May 2021, six unrelated persons traveling from Nigeria received diagnoses of monkeypox in non-African countries: four in the United Kingdom and one each in Israel and Singapore. In July 2021, a man who traveled from Lagos, Nigeria, to Dallas, Texas, became the seventh traveler to a non-African country with diagnosed monkeypox. Among 194 monitored contacts, 144 (74%) were flight contacts. The patient received tecovirimat, an antiviral for treatment of orthopoxvirus infections, and his home required large-scale decontamination. Whole genome sequencing showed that the virus was consistent with a strain of *Monkeypox virus* known to circulate in Nigeria, but the specific source of the patient's infection was not identified. No epidemiologically linked cases were reported in Nigeria; no contact received postexposure prophylaxis (PEP) with the orthopoxvirus vaccine ACAM2000.

## Findings

On July 13, 2021, an emergency department (ED) physician in Dallas evaluated an early middle-aged man* with a 2-week history of fever, cough, and fatigue, followed by onset of a diffuse rash. Less than 1 week earlier, the patient had been in Nigeria for a large social gathering. Because of the extensive pustular rash on his face, hospital staff members immediately placed the patient in an airborne isolation room, where he was managed with airborne and contact precautions plus eye protection. After reviewing CDC’s Travelers’ Health destination webpage for Nigeria,^†^ the ED physician suspected monkeypox, and public health authorities were immediately notified. The following day, the Dallas County Health and Human Services Laboratory Response Network laboratory confirmed, by real-time polymerase chain reaction (PCR), the presence of nonvariola orthopoxvirus DNA from lesion swabs. Subsequent testing by species-specific real-time PCR at CDC confirmed West African clade *Monkeypox virus*.

Interviews revealed that the patient had arrived in Nigeria on June 25 and stayed in three urban centers during his trip (Figure). By June 30, he began experiencing diarrhea, vomiting, cough, subjective fever, and fatigue, all characteristic signs and symptoms of the monkeypox prodrome, which also mark the onset of transmissibility of the virus to others (e.g., through infected body fluids or respiratory droplets). On July 8, 1 day before boarding the first of two return flights, the patient developed a purulent rash confined to a covered part of his body. After a brief layover in the Atlanta airport, he took a domestic flight to Dallas, and then a ride-share vehicle to his residence, where he lives alone. The next day, the rash had worsened and was visible on his face, prompting a friend to drive him to the hospital on July 13. Like many persons his age, the patient had never received the smallpox vaccine, which would have provided cross-protection against monkeypox (*2*) but has not been routinely administered following the eradication of smallpox in 1980 (*3*).

**FIGURE:**
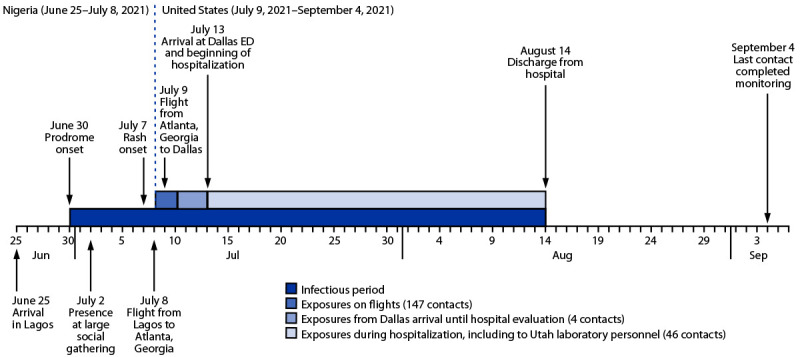
Time line of patient activities and potential exposures to *Monkeypox virus* from patient’s arrival in Lagos, Nigeria to completion of monitoring for the last exposed known contact — Dallas, Texas, June–September 2021 **Abbreviation:** ED = emergency department.

## Public Health Response

CDC, state and local public health authorities, and the treating clinicians launched an intensive investigation during July 13–September 4. Investigators reviewed what is known about orthopoxviruses and, through iterative discussions, categorized exposures as high, intermediate, low/uncertain, or no risk (Table). Exposures were ascertained through information collected from airport video surveillance, the patient’s report of his activities and interactions with others, and flight seating assignments. This activity was reviewed by CDC and was conducted consistent with applicable federal law and CDC policy.^§^

**TABLE:** Recommendations for monitoring and postexposure prophylaxis with the orthopoxvirus vaccine ACAM2000,* by risk level of exposure to a monkeypox patient during the period of interest^†^ (N = 223 contacts)^§^ — Dallas, Texas, July 2021

Exposure risk level	Recommendations		Exposure characteristic	Specific population for this event	No. of persons monitored/Total no. (%)
	Monitoring^§^	PEP^¶^			
High	Monitoring	Recommended	Contact between a person’s broken skin or mucous membranes and the materials,** skin, lesions, or body fluids from patient (e.g., saliva from patient inadvertently splashes eye or oral cavity of a person)	NA	0 (—)
			Presence near patient during aerosol-generating procedure (e.g., intubation) while not wearing a surgical face mask or respirator		
			Exposure that, at the discretion of public health authorities, was recategorized to this risk level (i.e., exposure that ordinarily would be considered a lower risk exposure, raised to this risk level because of unique circumstances)		
Intermediate	Monitoring	Might be recommended after consultation with public health authorities and consideration of the risks and benefits	Contact between a person’s intact skin and the materials,** skin, lesions, or body fluids from patient	Friend who visited patient and touched patient’s used or potentially soiled clothing	1/1 (100)
				Flight crew who provided service to patient and had opportunities for direct contact with patient materials** (e.g., handling of used drinking cups or improper doffing of gloves)	6/7 (86)
			Presence ≤6 ft of patient for >3 hrs	Passengers seated ≤6 ft of patient during the Lagos to Atlanta flight	21/23 (91)
			Care provided to patient by health care provider not wearing gown, gloves, eye protection, and N95 or other respirator on one or more occasions	NA	0 (—)
			Exposure that, at the discretion of public health authorities, was recategorized to this risk level because of unique circumstances (e.g., if the potential for an aerosol exposure is uncertain, public health authorities may choose to decrease risk level from high to intermediate)	Laboratory personnel ≤6 ft from a laboratory instrument that had the potential for aerosol generation during analysis of patient specimens	3/3 (100)
Low/ Uncertain	Monitoring	None	Care provided to patient by a health care provider who, during all interactions, wore gown, gloves, eye protection, and N95 or other respirator	Health care provider who cared for patient	43/43 (100)
			Exposure that, at the discretion of public health authorities, was recategorized to this risk level based on unique circumstances (e.g., uncertainty about whether *Monkeypox virus* was present on a surface and uncertainty about whether a person touched that surface)	Passengers on the international flight who might have used the mid-cabin lavatory used by patient	112/138 (81)
				Passengers on the domestic flight, seated adjacent to patient with potential for contact with patient or contaminated materials** because of narrow space (e.g., sharing armrest)	5/5 (100)
				Ride-share driver of an enclosed vehicle who drove patient while both wore cloth masks	1/1 (100)
				Friend who visited patient’s home, but denied contact with any surfaces or with patient	1/1 (100)
				Unspecified community contact	1/1 (100)
No risk	None	None	Exposure that public health authorities deemed did not meet criteria for other risk categories	Example: Customs and Border Protection officers who briefly handled patient materials (e.g., passport)** while wearing gloves	0 (—)
				Example: persons at gate of an airport at the same time as patient	
				Example: cleaners of mid-cabin lavatory of international flight and janitorial staff in airport bathrooms who were confirmed to have worn eye protection, gloves, gowns, and surgical masks	

**Abbreviations:** NA = not applicable; PEP = postexposure prophylaxis.

* https://www.fda.gov/vaccines-blood-biologics/vaccines/acam2000-smallpox-vaccine-questions-and-answers

^†^ Period of interest was from onset of prodromal symptoms through resolution of the rash (i.e., shedding of crusts and observation of healthy pink tissue at all former lesion sites).

^§^ Monitoring includes ascertainment of selected signs and symptoms of monkeypox: fever (≥100.4°F [≥38°C]), chills, new lymphadenopathy (periauricular, axillary, cervical, or inguinal), and new skin rash ≥21 days after the exposure to the patient or the patient’s materials. Monitoring could involve in-person visits, regular communications (e.g., phone call or another system) between public health representatives and the person under monitoring, self-monitoring by persons and reporting of symptoms to health departments only if symptoms appear, or another reliable system determined by the health department. Health departments should take into consideration the person’s exposure risk level, the number of persons needing monitoring, the time since exposure, and the available resources, when determining the type of monitoring to be conducted. Persons should be advised to self-isolate if any symptoms develop. Persons who report only chills or lymphadenopathy should remain at their residence, self-isolate for 24 hours, and monitor their temperature for fever; if fever or rash do not develop and chills or lymphadenopathy persist, the person should be evaluated by a clinician for the potential cause. Clinicians can consult with the state health department if monkeypox is suspected. If a fever or rash develops, CDC should immediately be consulted.

^¶^ During this investigation, no contacts received PEP with ACAM2000.

** Linens, health care equipment used for patient care, surfaces potentially soiled by patient, and personal items belonging to patient.

Notifications were immediately issued to the public via press releases; clinicians through the Health Alert Network; country national focal points, including those in Nigeria, under guidelines of the *International Health Regulations (2005)*; public health officials via an Epi-X^¶^; and a call with state health departments. Airline and federal partners provided information for flight contacts. Simultaneously, investigators discussed disinfection of potentially contaminated surfaces. Transmission to passengers on subsequent flights was considered unlikely because the disinfectants used between flights included a label claim for inactivation of *Vaccinia virus*, suggesting effective inactivation of *Monkeypox virus*. Airport bathrooms used by the patient were confirmed to have been regularly cleaned with similarly effective products. The owners of the two cars used by the patient were instructed to disinfect high-touch surfaces in the car with such products.

A total of 223 contacts were identified. A 24/7 CDC monkeypox call center was established to coordinate daily monitoring from 21 U.S. jurisdictions where contacts resided and to provide clinical consultations to physicians who suspected additional cases. Recommendations about monitoring and PEP were strongest for persons with high (no persons) or intermediate (34 persons) risk. Some exposures were categorized or recategorized to a higher or lower risk level because of circumstances unique to this event. For example, typically, persons who used the same lavatory as the patient would be categorized as no risk; however, because the patient used the mid-cabin aircraft lavatory while he had a purulent rash on his body, investigators included passengers who used that same lavatory in the “low/uncertain” risk group. Similarly, persons seated adjacent to the patient on the domestic flight (<3 hours) had increased opportunities for contact with the patient’s skin or contaminated materials (e.g., shared arm rest); however, because it was uncertain whether this exposure had occurred, investigators categorized these as “low/uncertain” risk. Reaching identified travel contacts was challenging; some travelers were non-U.S. residents, had already departed the United States, or had provided inaccurate telephone numbers to airlines, all of which hindered timely contact tracing.

Approximately 1 week after the investigation began, investigators learned that early in the patient’s hospitalization, clinical specimens from the patient had been sent from the hospital to a laboratory in Utah for additional diagnostics. Conversations with the laboratory’s biosafety manager revealed that instrumentation used to process these specimens during July 13–19 might have resulted in aerosol generation outside of a biosafety cabinet where personnel were not wearing respirators; six laboratory personnel were accordingly added to contact monitoring.

Most (189; 85%) contacts were categorized as having “low/uncertain” risk. All monitoring of known contacts concluded on September 4, 2021, and no secondary cases in the United States were identified, including among persons with suspected cases reported by clinicians to the CDC call center. ACAM2000, the orthopoxvirus vaccine recommended after exposure to smallpox (*4*), might be helpful after exposure to *Monkeypox virus* and was available for this potential indication through a CDC Investigational New Drug Protocol. Local public health officials were recommended to consider ACAM2000 for the 34 contacts with intermediate risk; no contact for whom ACAM2000 was offered received the vaccine. The patient completed a 32-day hospitalization that included treatment with tecovirimat because of severe disease. Hospital discharge had been delayed until a remaining lesion tested negative for *Monkeypox virus* DNA by PCR; this ensured no infectious risk upon discharge but extended the hospitalization. Procedures for disinfection of his home were extrapolated from guidance previously developed by CDC for smallpox** and by Public Health England for monkeypox.^††^ Whole genome sequencing confirmed that the *Monkeypox virus* strain was closely related to strains known to circulate in Nigeria. No specific source of his infection was identified in Nigeria; local staff members dispatched to the urban centers visited by the patient found no reported cases linked to him.

## Discussion

This was the first travel-associated monkeypox case in the United States, and the seventh such case worldwide, since a large 2017 outbreak in Nigeria (*5*,*6*). Case recognition launched a large public health response involving extrapolation of limited data about monkeypox to develop a framework for managing potentially exposed persons and preventing additional cases.

The reservoir for *Monkeypox virus* has not been identified but is suspected to be a rodent or other small mammal or mammals (*2*). Before the 2017 Nigeria outbreak, most human monkeypox cases occurred in rural, forested areas of Africa; however, in Nigeria, monkeypox cases have occurred in urban areas, suggesting novel risk factors (*1*). Two distinct clades of *Monkeypox virus* circulate in Africa: the West African clade, which is endemic in west Africa, and the Congo Basin clade, which occurs in central Africa (*2*). Complicating the public health response, cases in Nigeria, although confirmed to be caused by the West African clade, have clinically been distinct: the West African Clade is historically believed to cause milder human disease, few deaths, and limited human-to-human transmission. However, some cases in Nigeria have been severe, even resulting in death, most commonly in persons with HIV infection (*1*,*7*). In addition, epidemiologic and genomic analyses have shown multiple human-to-human transmission events in Nigeria, including within households and a prison; secondary cases occurred in a health care provider and in family members of one patient whose illness was diagnosed in the United Kingdom (*1*,*7*,*8*).

Fortuitously, mask use during the ongoing COVID-19 pandemic ensured that contacts, including fellow airline passengers and crew members, community contacts, and health care providers, were at reduced risk for being infected with *Monkeypox virus* from this patient. Sparse data on *Monkeypox virus* epidemiology, increasing numbers of immunocompromised persons in the United States (*9*) (e.g., from chemotherapy and other therapeutics), and waning of immunity to orthopoxviruses since the eradication of smallpox and cessation of routine smallpox vaccination (*2*) led investigators in this situation to take a cautious approach. Clinical laboratory personnel are typically not at risk for exposure to *Monkeypox virus*; however, use of specific laboratory instruments near persons not wearing adequate personal protective equipment caused investigators to be concerned about exposure to aerosols. The possible presence of *Monkeypox virus* on the patient’s covered body during lavatory use similarly prompted a guarded approach; passengers who used that lavatory were monitored in the “low/uncertain” risk group. Most exposures occurred during airline flights that, relative to the time the patient was in the community and admitted to the hospital, were brief.

Four months after this case, an eighth travel-associated monkeypox case in a traveler from Nigeria occurred, also in the United States, prompting CDC to issue a Level 1 (Watch) Travel Health Notice for travel to Nigeria.^§§^ Multiple reasons have been proposed for continued human cases in Nigeria, including population growth, increased human interaction with *Monkeypox virus* reservoirs because of deforestation and climate change, accumulation of unvaccinated cohorts, and declining smallpox vaccine immunity (*1*,*10*). The Nigerian Federal Ministry of Health continues to work to prevent, detect, and investigate monkeypox cases in Nigeria, but as cases continue to occur, U.S. public health and hospital authorities might consider developing local strategies for responding to future imported cases. Early clinical suspicion facilitated by elicitation of a complete travel history, use of appropriate infection control precautions, and timely identification of activities performed during the period of infectivity were among the most critical actions taken. Understanding the types of exposures that are most concerning for *Monkeypox virus* transmission, knowing contaminated surfaces need to be quickly identified and decontaminated, and anticipating potentially long hospitalizations and contact monitoring periods might also aid in planning for future cases (Box).

BOX **Critical actions taken during response to an imported case of monkeypox in the United States and lessons learned — Dallas, Texas, July 2021**

- Elicitation of past-month travel history as part of every initial patient encounter in the treating hospital’s ED; facilitated expedited clinical diagnosis and isolation of the patient.
- Assurance of airborne and contact precautions, plus eye protection*; minimal numbers of staff members entering patient’s room; and log of all persons entering rooms ensured limited exposures and a record of persons to be monitored.
- Immediate notification of clinical suspicion to the local health department^†^ as part of the treating hospital’s ED protocol for situations such as this; enabled swift public health investigation.
- Consultation with CDC about antivirals and clinical management needs^§^; can be helpful early in the clinical course, particularly for severe infections.
- Collaboration with local LRN laboratory facilitated earliest testing to confirm that specimens from the patient had a DNA signature consistent with nonvariola orthopoxvirus; in the United States, this testing only possible at LRN laboratories and CDC.
- Identification of patient's period of infectivity; enabled identification of potential contacts during period of interest,^¶^ but required iterative interviews at multiple time points with patient.
- Establishment of local capacity to monitor contacts for 21 days from last exposure; was challenging, particularly with public health authorities strained by ongoing COVID-19 pandemic. Successful monitoring often involved providing case-by-case guidance (e.g., about delaying international travel plans) and negotiating with exposed persons regarding strategy that was least disruptive.
- Consideration of patient as infectious until all lesions are fully healed was safest approach; resulted in protracted hospitalization that local health authorities and treating hospitals should plan for.**
- Patient unwillingness, because of privacy concerns, to share details that would have aided the public health investigation in Nigeria hindered identification of infection source; patient trust, including in international authorities, is essential to determining infection source.
- Multiple communal settings identified for rapid decontamination despite the relatively short period between the patient’s flight and his hospitalization; common over-the-counter cleaning products with label claim for *Vaccinia virus* used for most surfaces, but additional steps needed in hospital and residence because of extensive contamination.
- Assurance by laboratories that specimens are handled according to the BMBL to prevent unintentional exposures.^††^

**Abbreviations:** BMBL = Biosafety in Microbiological and Biomedical Laboratories; ED = emergency department; LRN = Laboratory Response Network.

* CDC recommends at least droplet precautions for monkeypox, but whenever possible, airborne precautions should be used out of an abundance of caution.

^†^ Early notification, even while other diagnoses are being considered, is critical to the investigation. Public health authorities can facilitate CDC consultation and laboratory confirmation; timely communication of suspicions is essential to timely decontamination of contaminated surfaces (e.g., airplanes) and to begin contact tracing.

^§^ Therapeutics stockpiled by the U.S. government for prevention and treatment of smallpox can be considered for patients with monkeypox; these are available through Investigational New Drug protocols at CDC. https://www.cdc.gov/smallpox/prevention-treatment/index.html

^¶^ Patients with monkeypox are infectious from the onset of prodromal symptoms until crusts separate and a fresh layer of skin forms underneath.

** Health care facilities and public health authorities should be familiar with the tiered U.S. Regional Treatment Network for special pathogens, including how to contact the Regional Ebola and other Special Pathogen Treatment Center for their jurisdiction for further consultation about persons with suspected or confirmed infection with a special pathogen. https://www.phe.gov/Preparedness/planning/hpp/reports/Documents/RETN-Ebola-Report-508.pdf

^††^ https://www.cdc.gov/labs/BMBL.html

**Summary**

**What is already known about this topic?**

Monkeypox is a rare, potentially serious zoonotic infection. During September 2018–June 2021, six cases among travelers from Nigeria to non-African countries were identified; two instances resulted in secondary cases.

**What is added by this report?**

In July 2021, *Monkeypox virus* was confirmed in a U.S. resident who had returned from Nigeria. The public health investigation included identifying and monitoring exposed persons and disinfecting potentially contaminated surfaces. No secondary cases occurred.

**What are the implications for public health practice?**

Continued *Monkeypox virus* transmission in Nigeria might lead to additional sporadic U.S. importations. Early clinical suspicion, prompt reporting to public health authorities, and awareness of the types of exposures that might be high risk are among the lessons learned.

## Footnotes

* Past medical history and identifying information withheld to protect patient confidentiality.

†
https://wwwnc.cdc.gov/travel/destinations/traveler/none/nigeria?s_cid%20=%20ncezid-dgmq-travel-single-001

§ 45 C.F.R. part 46, 21 C.F.R. part 56; 42 U.S.C. Sect. 241(d); 5 U.S.C. Sect. 552a; 44 U.S.C. Sect. 3501 et seq.

¶
https://emergency.cdc.gov/epix/index.asp

**
https://biotech.law.lsu.edu/blaw/bt/smallpox/CDC-28feb2010/index.htm

††
https://www.gov.uk/guidance/monkeypox#guidance-for-environmental-cleaning-and-decontamination

§§
https://wwwnc.cdc.gov/travel/notices/watch/monkeypox-nigeria
